# Twelve Weeks of a Staged Balance and Strength Training Program Improves Muscle Strength, Proprioception, and Clinical Function in Patients with Isolated Posterior Cruciate Ligament Injuries

**DOI:** 10.3390/ijerph182312849

**Published:** 2021-12-06

**Authors:** Cheng-Chang Lu, Hsin-I Yao, Tsang-Yu Fan, Yu-Chuan Lin, Hwai-Ting Lin, Paul Pei-Hsi Chou

**Affiliations:** 1 Department of Orthopaedic Surgery, Kaohsiung Municipal Siaogang Hospital, Kaohsiung 812, Taiwan; cclu0880330@gmail.com; 2 School of Medicine, College of Medicine, Kaohsiung Medical University, Kaohsiung 807, Taiwan; 3 Department of Orthopaedic Surgery, Kaohsiung Medical University Hospital, Kaohsiung 807, Taiwan; 910095@ms.kmuh.org.tw; 4 Regenerative Medicine and Cell Therapy Research Center, Kaohsiung Medical University, Kaohsiung 807, Taiwan; 5 Kaohsiung Municipal Kaohsiung Commercial High School, Kaohsiung 800, Taiwan; Pokeryao11@gmail.com; 6 Department of Sports Medicine, Kaohsiung Medical University, Kaohsiung 807, Taiwan; littlefan03@gmail.com (T.-Y.F.); whiting@kmu.edu.tw (H.-T.L.); 7 Doctoral Degree Program in Biomedical Engineering, Kaohsiung Medical University, Kaohsiung 807, Taiwan

**Keywords:** posterior cruciate ligament, balance training, Lysholm score, IKDC, proprioception

## Abstract

Ligament reconstruction is indicated in patients with an isolated posterior cruciate ligament (PCL) injury who fail conservative treatment. To eliminate the need for PCL reconstruction, an ideal rehabilitation program is important for patients with an isolated PCL injury. The purpose of this study was to investigate the improvement in functional outcome, proprioception, and muscle strength after a Both Sides Up (BOSU) ball was used in a balance combined with strength training program in patients with an isolated PCL injury. Ten patients with isolated PCL injuries were recruited to receive a 12 week training program as a study group. In the control group (post-PCL reconstruction group), ten subjects who had undergone isolated PCL reconstruction for more than 2 years were enrolled without current rehabilitation. The Lysholm score, IKDC score, proprioception (active and passive), and isokinetic muscle strength tests at 60°/s, 120°/s, and 240°/s, were used before and after training on the injured and normal knees in the study group, and in the post-PCL reconstruction group. The results were analyzed with a paired *t*-test to compare the change between pre-training, post-training, and the normal leg in the study group, and with an independent *t*-test for comparisons between the study and post-PCL reconstruction groups. Both the Lysholm and IKDC scores were significantly improved (*p* < 0.01) after training, and no difference was observed compared to the post-PCL reconstruction group. The active and passive proprioception was improved post-training compared to pre-training, with no difference to that in the post-PCL reconstruction group. Isokinetic knee quadriceps muscle strength was significantly greater post-training than pre-training in PCL injured knees at 60°/s, 120°/s, and 240°/s, and in hamstring muscle strength at 60°/s and 120°/s. Muscle strength in the post-training injured knee group showed no significant difference compared to that in the post-training normal leg and the post-PCL reconstruction group. The post-training improvement of muscle strength was higher in the PCL injured leg compared to the normal leg and there was no difference between the dominant and non-dominant injured leg in the study group. After 12 weeks of BOSU balance with strength training in patients with an isolated PCL injury, the functional outcome, proprioception, and isokinetic muscle strength were significantly improved, and comparable to the contralateral normal leg and the post-PCL reconstruction group. We suggest that programs combining BOSU balance and strength training should be introduced in patients with a PCL injury to promote positive clinical results.

## 1. Introduction

The main function of the posterior cruciate ligament (PCL) is to restrict tibial posterior translation and provide a secondary restraint to tibial rotation [1,2,3]. The reported incidence of PCL injury is 1%–44%, which is rare compared to anterior cruciate ligament injury [4,5,6,7,8]. PCL injury occurs more frequently in polytraumatized patients combined with other associated knee injuries [6,9,10]. Generally, nonoperative treatment is indicated for patients with isolated grade I or II PCL injuries, or those with grade III injuries but who have mild symptoms or low activity demands [4,7,8,11]. However, patients with chronic PCL injuries often sustain anterior knee pain, the sensation of instability, difficulty in ascending stairs, and an inability to bear weight [12,13,14]. In addition, chronic PCL deficiency changes knee kinematics and loads with increasing contact stress over the medial and patellofemoral compartments, resulting in osteoarthritis changes and damage to the meniscus and posterolateral structures [15,16,17,18,19].

A specific rehabilitation program is important for patients with an isolated PCL injury to improve knee biomechanics, decrease knee load, reduce the possibility of degenerative change, and allow an early return to sport. In contrast to the comprehensive training program proposed for an ACL injury [20,21,22], few scholars have reported training programs for patients with a PCL injury. The general principles of rehabilitation for patients with isolated PCL injuries are that they should avoid posterior tibial translation in the early injury period to enhance ligament healing, and this should be followed by a progressive range of motion and strengthening of the quadriceps and core musculature [23]. Therefore, the restoration of muscle power and proprioception is important for PCL knee injury recovery [24]. However, ideal training rehabilitation strategies remain undetermined.

Balance training has been demonstrated to enhance knee proprioception, resulting in improved leg stability, dynamic postural control, sports performance, and the prevention of knee injuries [22,25,26,27,28]. Several studies have demonstrated that strengthening combined balance training restores better functional recovery compared to strength training alone [20,21]. Eitzen et al. [20] introduced a 5-week combined strength and perturbation training program in patients with ACL injuries. The study results showed a significant improvement in knee function, muscle strength, and single-leg hop performance after training compared to pre-training. Hartigan et al. [21] investigated the effect of a training program with quadriceps strengthening combined with perturbation and compared it to a strengthening only program in non-copers with acute ACL injury. They found that the strengthening with perturbation training group presented more symmetric quadriceps muscle strength and knee excursions than the quadriceps strengthening only group. Paterno et al. [27] applied 6 weeks of strengthening and balance training with a Both Sides Up ball (BOSU) in high school athletes. They found significant improvements in single-limb total stability and anterior–posterior stability after training.

The purpose of this study was to evaluate the effect of BOSU balance training combined with muscle strengthening for 12 weeks in symptomatic isolated PCL injury patients. We hypothesized that in patients with an isolated PCL injury the training would significantly improve their functional outcomes, proprioception, and muscle strength; be responsible for a decrease in bilateral leg asymmetry around the normal leg, and produce results comparable to those in the post-PCL reconstruction group.

## 2. Materials and Methods

### 2.1. Subjects

This study evaluated the effect of 12 weeks of combined BOSU balance and muscle strength training in isolated PCL injury patients symptomatic in daily activities, during their clinic follow-ups. The inclusion criteria for the study group were as follows: (1) isolated PCL injury confirmed with MRI, (2) positive posterior drawer test, (3) injury time > 3 months without surgery, (4) symptomatic in daily activities, and (5) no other injuries in the bilateral lower legs. To test the results of the rehabilitation training compared to that after PCL reconstruction, this study recruited subjects who had previously undergone PCL reconstruction as the control group. In the post-PCL reconstruction group, the inclusion criteria included subjects who: (1) had undergone isolated PCL reconstruction for more than 2 years, (2) had no other injuries in the lower leg receiving PCL reconstruction or the contralateral leg, and (3) could undertake daily activity without restriction.

This study was approved by the university’s Institutional Human Subjects Review Board (IRB KMUHIRB-E(II)20170181). The subjects in the study group provided informed consent before the rehabilitation training. In the PCL reconstruction group, informed consent was provided and signed without current rehabilitation training.

### 2.2. Balance Combined with Strength Training Program

Each subject in the study group received a training program of 1 h per session, 2 times a week for 12 weeks. The program was divided into three sequential phases: initial phase (1st–4th week), intermediate phase (5th–8th week), and late phase (9th–12th week). The total training course included a warm-up (stationary bicycle, 15 min), muscle strengthening (20 min), BOSU balance training (15 min), and a post-training stretch (10 min).

#### 2.2.1. Warm Up

The subject performed stationary bicycle training with an intensity of 70 rpm for 15 min.

#### 2.2.2. Muscle Strength Training

The muscle strengthening program included knee extension and knee curl training. The muscle strength training intensity was 70% of 1RM, and consisted of 2 sets, with 12 repetitions each. The muscle strength test was performed on the 1st day of the 1st, 5th, and 9th week to determine the training intensity at that stage.

#### 2.2.3. Balance Training

Balance training was designed according to Paterno [27]. It was performed with 2 sets (hold for 1 min/set for 15 min) at a time, twice a week.

*Initial phase* 

**Figure 1:**
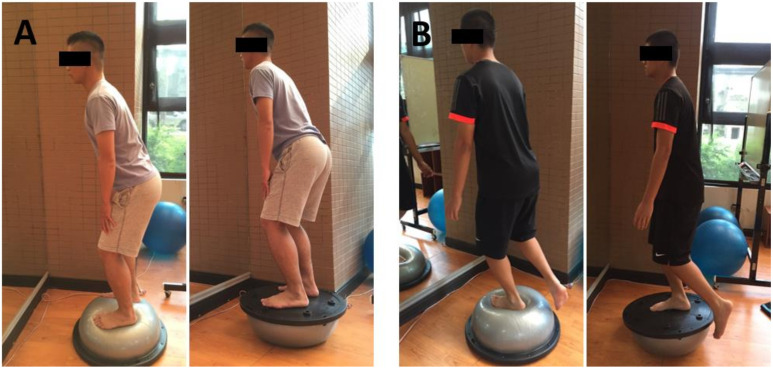
(**A**) Balance training in the initial phase. Subject performed bilateral leg stance exercises on the BOSU balance device. (**B**) Balance training in the late phase. Subject performed single leg stance exercises on the BOSU balance device.

The initial stage focused on whole-body balance and double-limb stability on an unstable surface using bilateral stance exercises on a BOSU balance device (DW Fitness, LLC, Madison, NJ, USA) (Figure 1A). BOSU is a device composed of a circular platform on one side and an inflated halfsphere on the other for stability and balance training.

*Intermediate phase* 

The intermediate phase was performed in the double-limb stance on the BOSU device with eyes closed to increase the challenge of the subject’s stability and balance.

*Late phase* 

The late phase was performed using a single-leg stance on the BOSU device to improve the single-leg stability (Figure 1B).

### 2.3. Posterior Cruciate Ligament Reconstruction Procedure

**Figure 2:**
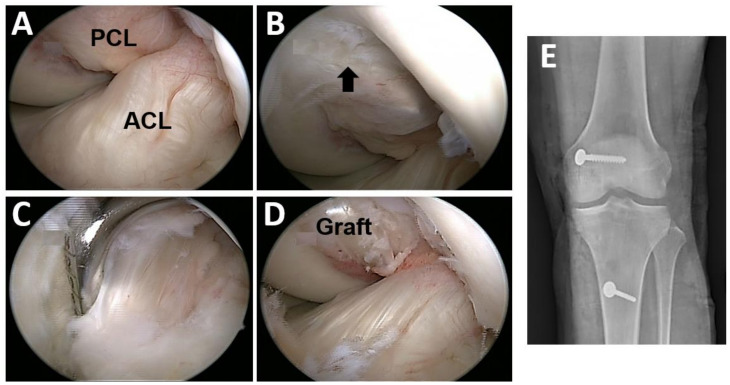
(**A**) Under arthroscopic examination, the posterior cruciate ligament was injured, and the anterior cruciate ligament showed pseudolaxity. (**B**) The femoral tunnel was created 6–8 mm away from the margin of the distal femoral condyle (black arrow). (**C**) The tibial tunnel was aimed by the guide at 10–12 mm under the joint surface (scope viewed through the posteromedial portal). (**D**) After graft fixation, the ACL restored its original tension. (**E**) The radiograph of the post-PCL reconstruction group. PCL: Posterior cruciate ligament. ACL: Anterior cruciate ligament.

In the post-PCL reconstruction group, we enrolled subjects who had undergone isolated PCL reconstruction for more than 2 years. All the PCL reconstruction operations were performed by the senior author (PH Chou) (Figure 2). In brief, the operator harvested the semitendinosus and gracilis tendons and prepared them to form a four-strand graft with a minimum 13 cm length. A femoral tunnel was created 6–8 mm from the anterior or distal medial femoral articular margin with the outside-in drill technique (Figure 2B), and the tibial tunnel was drilled from anterior to posterior about 10–12 mm below the joint line, aimed by the guide and under direct vision through the posteromedial portal (Figure 2C). After the graft passed the bone tunnels, the graft in the femoral tunnel was fixed first with one interference screw (Smith and Nephew, Andover, Massachusetts) and one cancellous post screw with a washer. Under an applied maximal anterior drawer force, the graft in the tibia tunnel was fixed with another interference screw and augmented with one cortical post screw with a washer (Figure 2E). After the operation, the patient was protected with a brace and crutches for 6 weeks.

### 2.4. Evaluations

#### 2.4.1. Functional Score Analysis

The subjects were evaluated for functional outcomes using the Lyholsm score [29,30] and the International Knee Documentation Committee (IKDC) score [31,32]. Evaluations were performed before and after 12 weeks of training in the study group and also after recruiting in the control group. The Lysholm knee score was graded as excellent (score 95–100), good (score 84–94), fair (65–83), or poor (<65). The IKDC form involves four main areas: subjective assessment, symptoms, range of motion, and ligament examination.

#### 2.4.2. Proprioception Test

The proprioception test was performed by the active reproduction of a passive position test (RPP) and passive reproduction of a passive position test using a Biodex System 3 Pro (Biodex Medical System, New York, NY, USA). All subjects were seated with a knee flexion of 90° with eyes blinded with masks, and the targeted angle was set at 45° knee flexion. For the active RPP, the subjects were asked to actively extend the knee to the target angle. For the passive RPP, the subject’s knee was passively extended by a machine with a velocity of 30°/s. The subjects were asked to press a switch when they felt that the target angle was achieved. The differences between the targeted angles and the angles reproduced by the subjects were recorded. The test was repeated three times each for the PCL injured and normal leg in the study group, and for post-PCL reconstruction knees in the control group. We recorded the value (degree) of proprioception measurement as the average of three tests.

#### 2.4.3. Laxity Examination

Knee ligament laxity was tested with the KT-1000 arthrometer [33] (Medmetric, Inc., San Diego, CA, USA) to measure the posterior translation of the tibia compared to the femur. The measurements were performed at 30° knee flexion. The laxity examination was tested three times in the injured knee of the study group before and after the training, and in the operated knee of the post-PCL reconstruction group. The value (mm) of laxity examination was recorded as the average of three tests.

#### 2.4.4. Strength Test

All subjects underwent quadriceps and hamstring muscle strength testing using a Biodex System 3 Pro (Biodex Medical System, New York, NY, USA). Testing consisted of the maximal isokinetic muscle strength (peak torque; PT) at velocities of 60°/s, 120°/s, and 240°/s with a rest time of 30 s between tests. The test was performed with the subject in a seated position with the knee range of motion from full extension (0°) to 90° flexion according to the manufacturer’s specifications. Each subject performed one test with 5 repetitions for a given angular velocity per second [34,35], and the highest PT value was recorded. The muscle strength value was recorded as the maximum PT and normalized by individual body weight to relative PT (N·m/kg) [36].

### 2.5. Statistical Analysis

For the change in functional score, proprioception, knee laxity, and muscle strength after training, a paired *t*-test was used to analyze the differences between pre-training and post-training in PCL injured knees, and between injured knees and the normal leg in the study group. An independent *t*-test was used to analyze between the study group and the post-PCL reconstruction group. The significance level was set at *p* < 0.05. All experimental data were calculated using SPSS version 20.0, for Windows (SPSS Inc. Chicago, IL, USA). Normality was tested by the Shapiro-Wilks method (outcome variables were tested for normality before performing statistical analyses). The effect size of pre–post changes within groups was estimated via the standardized response mean, with mean differences between post-training and pre-training divided by the standard deviation of the difference scores. Data are presented as the mean (standard deviation, SD).

## 3. Results

### 3.1. Subject Profiles

**Figure 3:**
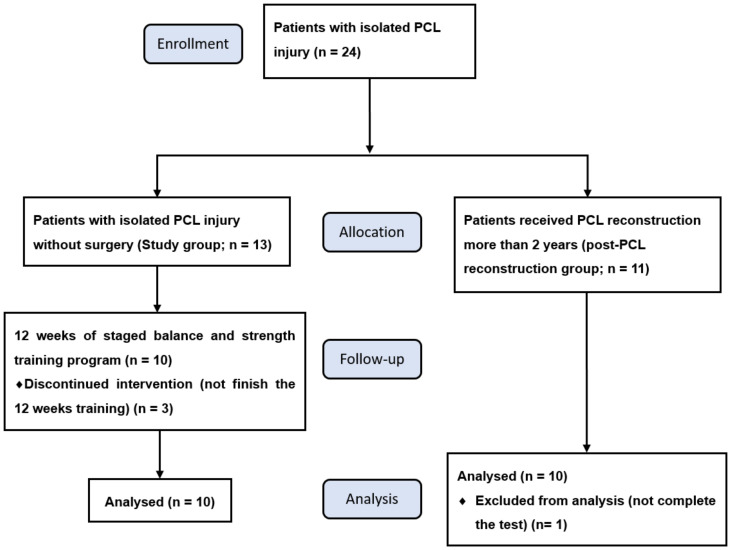
Flowchart of participant selection, follow-up and analysis. PCL, posterior cruciate ligament.

At the beginning of this study, 13 subjects in the study group and 11 subjects in the control (post-PCL reconstruction) group participated. Three subjects in the study group were withdrawn due to not finishing the 12-week rehabilitation program, and one subject in the control group left without completing the test (Figure 3). Finally, ten subjects were recruited in the study training group which included seven males and three females, and the 10 subjects in the post-PCL reconstruction group included seven males and three females. We calculated for the sample size of 10 paired patients, the assumption of a mean functional score difference (e.g., Lysholm score, pre-training to post-training) in the paired group was 22.00 points (SD 10.00) with a 0.40 correlation coefficient, which provided >90% power to detect such a difference with a paired *t*-test, with a two-sided Type I error of 0.05.

In the study group, five subjects injured their right knee PCL and five sustained left knee injuries. In the post-PCL reconstruction group, four subjects underwent PCL reconstruction in the right knee and six in the left knee. No patient presented with bilateral knee injuries in either group. In the study group, five subjects injured their dominant knee and five injured the non-dominant leg. There were no significant differences in age, body height, body weight, thigh circumference, initial knee range of motion and the level of daily physical activity (Tegner Activity Scale) [29,36] between the study group (before PCL injury) and the post-PCL reconstruction group (before test). After the end of 12 weeks training, the subjects in the study group declared that they could return to pre-injury physical activity. The subject profiles are shown in Table 1. All experimental data is summarized in Table 2.

### 3.2. Functional Outcome and Proprioception Were Significantly Improved after BOSU Balance and Strength Training in Patients with an Isolated PCL Injury

#### 3.2.1. Functional Score

**Figure 4:**
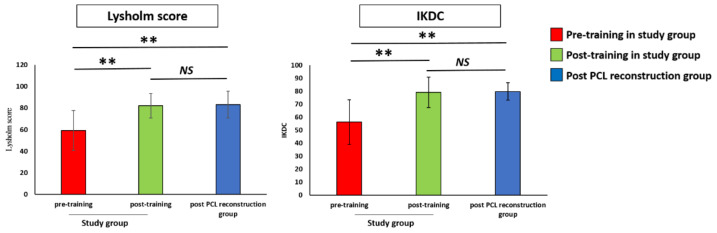
Comparison of Lysholm and IKDC scores at pre-training, at post-training, and in the post-PCL reconstruction group. IKDC: International Knee Documentation Committee. PCL: Posterior cruciate ligament. ** *p* < 0.01 indicates high statistical significance between two groups. NS: No significant difference between two groups.

The mean Lysholm score of the pre-training group (59.30 ± 19.49) was significantly lower than that in the post-PCL reconstruction group (83.20 ± 13.18, *p* < 0.01), and the score significantly improved after training (82.20 ± 11.94, *p* < 0.01) at the last follow-up. The mean IKDC score in the pre-training group (56.30 ± 18.07) was significantly lower than that in the post-PCL reconstruction group (79.90 ± 7.20, *p* < 0.01), and the score increased significantly to 79.20 ± 12.40, post-training at the last follow-up (*p* < 0.01). After training, the mean Lysholm and IKDC scores of the study group showed no significant differences from those of the post-PCL reconstruction group. The results of the functional scores in the study group and post-PCL reconstruction group are shown in Figure 4.

#### 3.2.2. Proprioception

**Figure 5:**
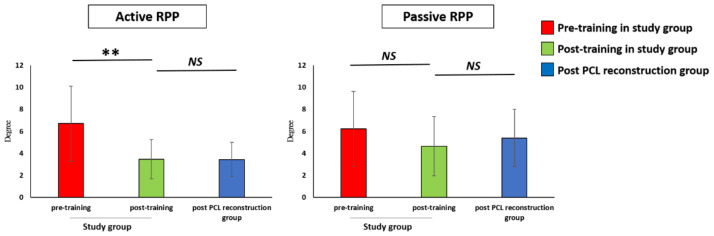
Comparison of proprioception change at pre-training, at post-training, and in the post-PCL reconstruction group. RPP: Reproduction of passive position test. PCL: Posterior cruciate ligament. ** *p* < 0.01 indicates high statistical significance between two groups. NS: No significant difference between two groups.

The active RPP was significantly improved (*p* < 0.01) after training (3.47 ± 1.89 °) compared to pre-training (6.70 ± 3.61 °). The passive RPP was improved post-training (4.66 ± 2.83 °) compared to pre-training (6.23 ± 3.56 °) without a significant difference. The results of the active (3.19 ± 1.46 °, *p* = 0.72) and passive (5.50 ± 2.85 °, *p* = 0.52) RPPs in the post-PCL reconstruction group showed no significant differences when compared to those of the post-training group. The results of the proprioception test are shown in Figure 5.

### 3.3. No Improvement in Knee Joint Laxity after BOSU Balance and Strengthening Training in Patients with an Isolated PCL Injury

#### Laxity Examination

The result of the laxity examination showed no significant difference in the post-training group (3.14 ± 0.93 mm) when compared to the pre-training (3.03 ± 0.61 mm, *p* = 0.82) and post-PCL reconstruction groups (3.10 ± 1.12 mm, *p* = 0.93).

### 3.4. Quadriceps and Hamstring Muscle Strength after Training Was Significantly Improved in the PCL Injured Knee and Reached the Level of the Post-Training Normal Leg and the Post-PCL Reconstruction Group

All subjects underwent a 60°/s, 120°/s, and 240°/s isokinetic muscle strength examination. Before training, the study group showed a decrease in the knee quadriceps and hamstring muscles strength in the PCL injured leg compared to their pre-training normal leg, with a significant difference in the isokinetic knee extension test at 120°/s and in the flexion test at 60°/s and 120°/s.

The knee quadriceps muscle strength significantly improved post-training compared to pre-training for all 60°/s (pre-training versus post-training; 1.01 ± 0.27 versus 1.40 ± 0.39 N·m/kg, *p* < 0.01), 120°/s (0.77 ± 0.26 versus 1.12 ± 0.23 N·m/kg, *p* < 0.01), and 240°/s (0.65.45 ± 0.12 versus 0.86 ± 0.13 N·m/kg, *p* = 0.01) tests. The quadriceps muscle strength in the post-training PCL injured leg achieved that of the post-training normal leg, and was significantly higher at 120°/s (*p* = 0.03). For the post-training PCL injured leg in the study group compared to the post-PCL reconstruction group, there were no significant differences for all 60°/s, 120°/s, and 240°/s tests.

After training, the knee hamstring strength in the PCL injured leg had significantly improved post-training compared to pre-training at 60°/s (0.46 ± 0.19 versus 0.71 ± 0.33 N·m/kg, *p* = 0.01) and 120°/s (0.44 ± 0.15 versus 0.62 ± 0.19 N·m/kg, *p* = 0.02). There was an increase in the knee hamstring muscle strength without statistical difference at 240°/s (0.50 ± 0.15 versus 0.60 ± 0.17 N·m/kg, *p* = 0.16) between pre-training and post-training in the PCL injured knee. On the other hand, the hamstring muscle strength in the post-training PCL injured leg reached the level of the post-training normal leg and that of the post-PCL reconstruction group for all 60°/s, 120°/s, and 240°/s isokinetic muscle strength examinations.

**Figure 6:**
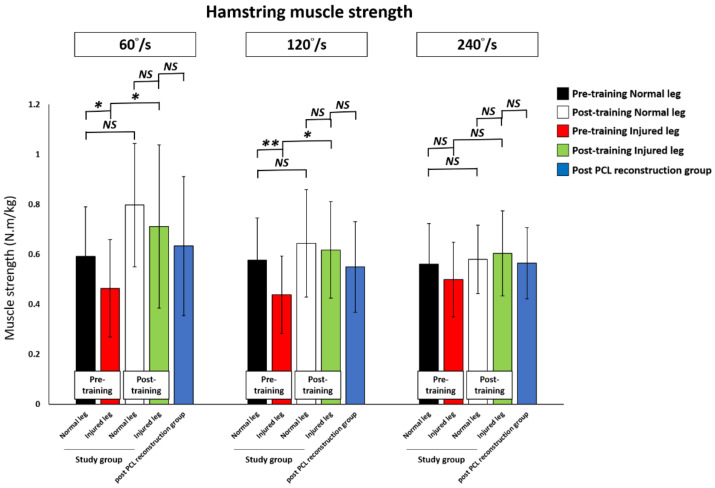
Comparison of the quadriceps muscle strength change at pre-training, at post-training, and in the post-PCL reconstruction group. PCL: Posterior cruciate ligament. * *p* < 0.05 indicates statistical significance. ** *p* < 0.01 indicates high statistical significance between two groups. NS: No significant difference between two groups.

**Figure 7:**
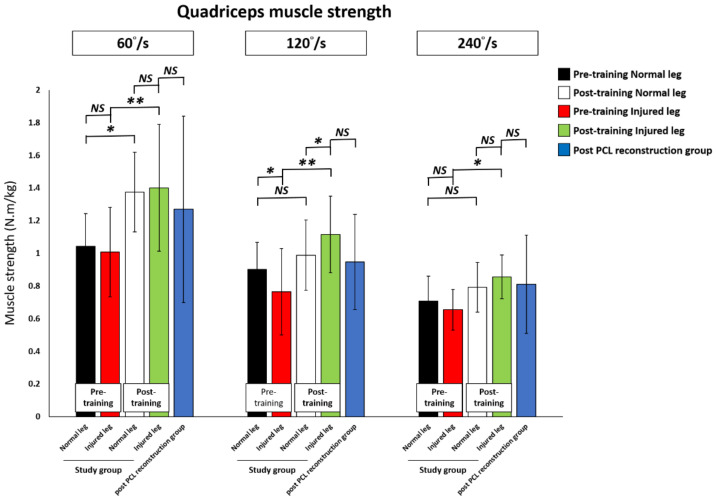
Comparison of hamstring muscle strength change at pre-training, at post-training, and in the post-PCL reconstruction group. PCL: Posterior cruciate ligament. * *p* < 0.05 indicates statistical significance. ** *p* < 0.01 indicates high statistical significance between two groups. NS: No significant difference between two groups.

The results of knee quadriceps and hamstring muscle strength changes in the study training group and the post-PCL reconstruction group are shown in Table 3, Figure 6 and Figure 7.

### 3.5. In the Study Group the PCL Injured Leg Presented a Higher Improvement in the Knee Muscle Strength Than the Normal Leg and there was No Difference between the Dominant and Non-Dominant Injured Legs

To investigate the rehabilitation effect on the muscle strength change in the patients with an isolated PCL injury after the 12 week staged balance and strength training program, we further analyzed the data between the normal and injured legs (Table 4) and between the dominant and non-dominant legs (Table 5) in the study group. The value change (post-training minus pre-training; N·m/kg) and improvement ((post-training minus pre-training)/pre-training; %) of the muscle strength were analyzed. In the study group, the value change of the knee muscle strength was higher in the injured leg compared that in the normal leg with a significant difference in the isokinetic muscle strength tests for extension at 120°/s (35.01% versus 8.67 %; *p* < 0.01), extension at 240°/s (20.15% vs 8.41%; *p* < 0.01) and flexion at 120°/s (17.94% versus 6.69 %; *p* = 0.0457). The improvement of the muscle strength in the PCL injured leg was higher than that of the normal leg in the isokinetic muscle strength tests, with a significant difference for extension at 120°/s (58.25% versus 13.80 %; *p* < 0.01), and extension at 240°/s (36.22% vs 16.73%; *p* < 0.01). The results of the muscle strength change and improvement are summarized in Table 4.

Comparing between the dominant and non-dominant injured legs in the study (training) group, we found that both the value change and improvement of knee muscle strength showed no significant difference between these two groups (Table 5).

## 4. Discussion

After a PCL injury, patients sustain persistent knee pain, weakness, and a sensation of instability, which impair daily and sports abilities. PCL reconstruction is indicated in patients with failed conservative treatment. In this study, we introduced a 12-week BOSU balance combined with strength training program for isolated PCL injury patients with the purpose of improving clinical activity, knee laxity, muscle strength, and eliminating the need for PCL reconstruction. The study results showed a significant improvement in the clinical functional score (Lysholm, IKDC) and proprioception after BOSU balance and strength training in patients with an isolated PCL injury. The knee quadriceps muscle strength at 60°/s, 120°/s, 240°/s, and hamstring muscle strength at 60°/s and 120°/s were significantly increased after training. At the same time, the functional score, proprioception, and quadriceps and hamstring muscle strength after training in the PCL injured knee were improved to the level of the post-training normal leg, and were comparable to those in patients who had undergone PCL reconstruction for more than 2 years. These findings indicate that combined BOSU balance and strength training could effectively enhance performance in injured knees and improve leg asymmetry in patients with isolated PCL injury, further decreasing the need for PCL reconstruction.

Patel et al. [4] investigated the natural history after an isolated PCL injury with a 6.9 year follow-up. In their study, the mean Lysholm-II knee score was 85.2 ± 10; it was excellent in 40%, good in 52%, fair in 3%, and poor in 5% of knees. The IKDC was 84 ± 12.3, with none of the patients presenting with normal results; 10% of patients had nearly normal results, 88% of patients had abnormal results, and 2% presented with severely abnormal results. Shelbourne et al. [8] reported their patients with an isolated, nonoperative PCL injury had a mean Lysholm score of 83.4. In this study, we recruited isolated PCL injury patients symptomatic in daily activity as a study group. Before training, the Lysholm score was 59.30 ± 19.49 and the IKDC score was 56.30 ± 18.07. These scores were lower than those in prior reports and lower than the post-PCL reconstruction group in this study. After training, the Lysholm score has significantly improved (*p* < 0.01) to 82.20 ± 11.94, with a grading of good. Similarly, the post-training IKDC score has significantly increased (*p* < 0.01) to 79.20 ± 12.40. Furthermore, both the Lysholm and IKDC scores in the post-training group were not significantly different from those of patients who had undergone PCL reconstruction. These findings indicated that knee function was significantly improved after 12 weeks of training in isolated PCL injury patients and achieved comparable functional results to patients who had undergone PCL reconstruction for more than 2 years.

In the knee joint, proprioceptive mechanoreceptors are important for joint sensation, movement, and rehabilitation [37]. The mechanoreceptors have been identified in the knee articular structures, that is, ACL, collateral ligaments, meniscus, and PCL [38,39]. After PCL injuries, investigators have found that knee proprioception significantly decreases compared to that in the healthy leg [24,37,40,41]. After 12 weeks of BOSU balance combined with strength training, both the active and passive RPP in the PCL injured knee had improved compared to pre-training and showed no significant differences compared to the post-PCL reconstruction group. The results indicated that combined balance and strength training enhanced proprioception in the PCL injured knee joint, which not only helps in returning to sports, but also reduces the possibility of further injury.

In the case of a PCL injury, the posterior tibial translation will increase beyond 30° of flexion compared to the contralateral intact knees. At a 90° flexion, PCL deficiency can increase posterior tibial translation by 3.5 mm (*p* < 0.05) [42]. However, the effect of muscle strengthening on decreasing the posterior translation in PCL injured knees is controversial. Furthermore, the relationship between the residual knee laxity and return to sports and the functional scoring is not conclusive. In this study, although the functional score improved after 12 weeks of BOSU balance combined with strength training, the results did not show a significant difference in the tibial displacement in the PCL injured knee before (3.03 ± 0.61 mm) and after (3.14 ± 0.93 mm) training. Similarly to our result, Shelbourne et al. [8] found that knee function restoration was independent of the degree of PCL laxity in athletically active patients who sustained a PCL injury and who were treated nonoperatively. Patel et al. [4] also reported in their findings that there was no significant association between the grade of PCL laxity and the Lysholm knee score.

After a PCL injury, patients sustain a decrease in lower leg muscle strength and atrophy due to pain and disuse [43,44,45]. Lee et al. [44] found decreased quadriceps (uninvolved versus involved, 203.6 ± 65.4 versus 129.9 ± 56.2 N-m) and hamstring muscle strength (101.4 ± 45.5 versus 45.8 ± 42.3 N-m) in a PCL injured group. Tibone et al. [45] also found a 20% decline in the quadriceps muscle in a 60°/s isokinetic test in patients with a PCL deficiency compared to the normal population. In this study, we also found that leg muscle strength asymmetry occurred in the patients with an isolated PCL injury. The study revealed a decrease in the quadriceps (significant difference at 120°/s) and hamstring (significant difference at 60°/s, 120°/s) muscle strength before training compared to the pre-training normal leg in the study group. After 12 weeks training, both quadriceps and hamstring muscle strength in the PCL injured leg showed comparable values to both the post-training normal leg in the PCL injured patients and the post-PCL reconstruction group. These findings demonstrated that both the quadriceps and hamstring muscles in the PCL injured knee were significantly stronger after the 12-week BOSU balance combined with strength training program, and the program improved the leg muscle strength asymmetry and achieved a comparable level to post-PCL reconstruction patients.

The rehabilitation effect on the improvement of knee muscle strength between the injured knee and uninvolved knee to decrease leg asymmetry in patients with knee cruciate ligament injury is another concern [34,36,46]. Czamara et al. [36] introduced a study to investigate the effect of the number and frequency of supervised physiotherapy visits on the knee muscle strength in patients after anterior cruciate ligament reconstruction (ACLR). They found that a lower number of visits in the ACLR group presented lower values of knee muscle strength in the operated leg compared to the uninvolved leg. On the other hand, a larger number of visits significantly improved the knee muscle strength in both the operated and involved knees. In another study, Czamara et al. [46] found that the isometric torque and peak torque of the tibial rotator muscle was significantly increased in patients after ACLR after 21 weeks of physiotherapy, and achieved the level of uninvolved knees and the normal population without knee injuries. In our study, we focused on the effect of a 12 week staged balance and strength training program on patients with an isolated PCL injury aiming to decrease the leg asymmetry between the injured leg and the normal leg. Our study results showed that the rehabilitation effect on the knee muscle strength was superior in the PCL injured leg compared to the normal leg in the study group. In addition, both the dominant and non-dominant PCL injured leg shared the same improvement effect on the quadriceps and hamstring muscle strength without a significant difference after 12 weeks of staged balance and strength training.

This study had some limitations. We investigated a limited number of cases with 10 patients with isolated PCL injuries and 10 subjects who had undergone PCL reconstruction. This study lacked sufficient case numbers to assign another group with strength training alone for comparison. In this study, only a 12-week-long training and evaluation program was investigated, so it was limited in terms of a long-term follow-up and maintaining the effect. Furthermore, there was no kinematic or kinetic study to investigate any changes in daily activities before and after training.

## 5. Conclusions

After 12 weeks of BOSU balance combined with muscle strengthening, patients with an isolated PCL injury demonstrated an improved clinical outcome, proprioception, and muscle strength, which decreased the leg muscle strength asymmetry and eliminated the need for PCL reconstruction.

## Figures and Tables

**Table 1:** Patient profiles in the study (training) and post-PCL reconstruction groups.

	Study Group	Post-PCL Reconstruction Group	*p* Value
Number (n)	10	10	
Gender (Male/Female)	7/3	7/3	
Injured site (Right/Left)	5/5	4/6	
Injured site (Dominant/Non-dominant)	5/5		
Age (years)	27.3 ± 9.4	30.1 ± 7.3	0.49
Body height (cm)	171.6 ± 6.5	169.8 ± 6.7	0.57
Body weight (Kg)	74.2 ± 11.9	72.4 ± 12.7	0.76
Thigh circumference (cm)	48.0 ± 3.5	46.6 ± 5.2	0.48
Total knee range of motion (degree)	118.7 ± 7.3	114.5 ± 7.4	0.24
Tegner Activity Scale	4.6 ± 1.7	4.9 ± 1.2	0.66

**Table 2:** Experimental data in the study (training) and post-PCL reconstruction groups.

	Study Group								Post-PCL Reconstruction Group	
	Pre-Training				Post-Training					
	Normal Leg	Shapiro -Wilk *p*	Injured leg	Shapiro -Wilk *p*	Normal Leg	Shapiro -Wilk *p*	Injured Leg	Shapiro -Wilk *p*	Post-PCL Reconstruction	Shapiro -Wilk *p*
Functional score										
Lysholm score			59.30 (19.49)	0.8377			82.20 (11.94)	0.1312	83.20 (13.18)	0.1378
IKDC			56.30 (18.07)	0.3701			79.20 (12.40)	0.7194	79.90 (7.20)	0.9777
Propriception (°)										
Active RPP			6.70 (3.61)	0.0784			3.47 (1.89)	0.9071	3.19 (1.46)	0.5020
Passive RPP			6.23 (3.56)	0.4584			4.66 (2.83)	0.1475	5.50 (2.85)	0.8414
Muscle strength (N·m/kg)										
Ext 60°/s	1.04 (0.20)	0.682	1.01 (0.27)	0.6784	1.38 (0.24)	0.5477	1.40 (0.39)	0.6746	1.27 (0.57)	0.4760
Ext 120°/s	0.90 (0.16)	0.705	0.77 (0.26)	0.0433	0.99 (0.21)	0.0635	1.12 (0.23)	0.7487	0.95 (0.29)	0.7761
Ext 240°/s	0.70 (0.15)	0.687	0.65 (0.12)	0.9340	0.79 (0.15)	0.2230	0.86 (0.13)	0.7566	0.81 (0.30)	0.0664
Flex 60°/s	0.59 (0.20)	0.313	0.46 (0.19)	0.9061	0.80 (0.25)	0.0321	0.71 (0.33)	0.6591	0.63 (0.28)	0.0803
Flex 120°/s	0.58 (0.17)	0.367	0.44 (0.15)	0.5667	0.64 (0.21)	0.3723	0.62 (0.19)	0.3855	0.55 (0.18)	0.1117
Flex 240°/s	0.56 (016)	0.012	0.50 (0.15)	0.4798	0.58 (0.14)	0.4771	0.60 (0.17)	0.6353	0.56 (0.14)	0.5260

Data are presented as the mean (standard deviation, SD). Normality was tested by the Shapiro-Wilks method (outcome variables were tested for normality before performing statistical analyses). RPP: Reproduction of passive position. Ext: Extension; Flex: Flexion.

**Table 3:** The analysis of knee quadriceps and hamstring muscle strength between the study (training) and post-PCL reconstruction groups.

	Analysis (*p*) of Quadriceps and Hamstring Muscle Strength Test					
Muscle Strength (N·m/kg)	Pre-Training Normal Leg vs. Pre-Training Injured Leg ^a^	Pre-Training Normal Leg vs. Post-Training Normal Leg ^a^	Pre-Training Injured Leg vs. Post-Training Injured Leg ^a^	Post-Training Injured Leg vs. Post-Training Normal Leg ^a^	Post-Training Injured Leg vs. Post-PCL Reconstruction ^b^	Effect Size (95% CI)
Ext 60°/s	0.6980	0.0250 *	0.0034 **	0.7995	0.5544	1.25 (0.68–1.81)
Ext 120°/s	0.0403 *	0.3770	0.0045 **	0.0261 *	0.1706	1.19 (0.65–1.73)
Ext 240°/s	0.0833	0.2542	0.0103 *	0.1245	0.6662	1.02 (0.56–1.49)
Flex 60°/s	0.0410 *	0.0832	0.0134 *	0.2202	0.5719	0.97 (0.53–1.41)
Flex 120°/s	0.0074 **	0.4477	0.0202 *	0.4965	0.4277	0.89 (0.49–1.29)
Flex 240°/s	0.0933	0.7964	0.1583	0.5025	0.5762	0.49 (0.27–0.71)

Effect size: pre–post changes within groups were estimated via the standardized response mean, with mean differences between post-training and pre-training divided by the standard deviation of the difference scores. An effect size > 0.80 is a large change. CI: Confidence interval. Ext: Extension; Flex: Flexion. ^a^ Analyzed by paired *t*-test. ^b^ Analyzed by independent *t*-test. * *p* < 0.05 indicates statistical significance. ** *p* < 0.01 indicates high statistical significance between two groups.

**Table 4:** Effects on the muscle strength change and improvement after training between the normal leg and PCL injured leg in the study group.

	Value Change of Muscle Strength in Study Group			Improvement of Muscle Strength in Study Group		
	Normal Leg (n = 10) (N·m/kg)	Injured Leg (n = 10) (N·m/kg)	*p* Value	Normal Leg (n = 10) (%)	Injured Leg (n = 10) (%)	*p* Value
Ext 60°/s	33.26 (12.40)	39.37 (9.99)	0.5385	38.86 (13.93)	43.16 (10.87)	0.7362
Ext 120°/s	8.67 (9.33)	35.01 (9.31)	0.0003 **	13.80 (12.15)	58.25 (17.07)	0.0010 **
Ext 240°/s	8.41 (6.91)	20.15 (6.23)	0.0074 **	16.73 (10.82)	36.22 (12.11)	0.0029 **
Flex 60°/s	20.5 (10.53)	24.73 (8.06)	0.6381	56.49 (26.95)	65.48 (25.43)	0.7289
Flex 120°/s	6.69 (8.43)	17.94 (6.37)	0.0457 *	17.72 (15.38)	50.79 (16.20)	0.300
Flex 240°/s	1.9 (7.15)	10.48 (6.81)	0.1732	10.79 (13.02)	28.67 (14.40)	0.1515

Data are presented as the mean (standard error, SE). The value change is calculated by post-training minus pre-training (N·m/kg). The improvement is calculated by (post-training minus pre-training)/pre-training, and the value is presented as percentage (%). * *p* < 0.05 indicates statistical significance. ** *p* < 0.01 indicates high statistical significance. Ext: Extension; Flex: Flexion.

**Table 5:** Effects on the muscle strength change and improvement after training between the dominant and non-dominant PCL injured leg in the study group.

	Value Change of Muscle Strength in Injured Leg in Study Group			Improvement of Muscle Strength in Injured Leg in Study Group		
	Dominant Leg (n = 5) (N·m/kg)	Non-Dominant Leg (n = 5) (N·m/kg)	*p* Value	Dominant Leg (n = 5) (%)	Non-Dominant Leg (n = 5) (%)	*p* Value
Ext 60°/s	40.12 (8.37)	38.62 (19.47)	0.9453	43.33 (9.33)	43.00 (21.09)	0.9891
Ext 120°/s	34.98 (8.83)	35.04 (17.65)	0.9973	57.05 (20.36)	59.45 (29.93)	0.9488
Ext 240°/s	23.78 (8.90)	16.52 (9.43)	0.4002	44.57 (19.18)	27.87 (16.02)	0.5233
Flex 60°/s	22.44 (11.37)	27.02 (12.68)	0.7299	41.35 (16.36)	89.61 (48.50)	0.3899
Flex 120°/s	18.96 (7.20)	16.92 (11.42)	0.7571	50.15 (18.30)	51.43 (29.07)	0.9714
Flex 240°/s	9.42 (10.05)	11.54 (10.35)	0.8314	23.67 (19.31)	33.67 (23.42)	0.7508

Data are presented as the mean (standard error, SE). The value change is calculated by post-training minus pre-training (N·m/kg). The improvement is calculated by (post-training minus pre-training)/pre-training, and the value is presented as percentage (%). Ext: Extension; Flex: Flexion.
